# Pulmonary Infection Caused by Mycobacterium malmoense and the Difficulties in Distinguishing It From the Pulmonary Mycobacterium tuberculosis Infection

**DOI:** 10.7759/cureus.59207

**Published:** 2024-04-28

**Authors:** Miwako Kogure, Takahiro Kaki, Yuhei Harutani, Koichi Sato, Kuninobu Kanai

**Affiliations:** 1 Department of Respiratory Medicine, Wakayama National Hospital, Hidaka, JPN; 2 Department of Respiratory Medicine, Naga Municipal Hospital, Kinokawa, JPN

**Keywords:** taqmanmtb, trcready, pulmonary infection, mycobacterium tuberculosis, mycobacterium malmoense

## Abstract

*Mycobacterium malmoense* is a rare species of non-tuberculosis mycobacteria detected in Japan that causes pulmonary infection and cervical lymphadenitis. Here, we report a case of pulmonary infection caused by *M. malmoense*, which was difficult to distinguish from *Mycobacterium tuberculosis* (Mtb) infection. A 64-year-old Japanese woman with a history of pulmonary* *tuberculosis had bloody phlegm, a cough, and discomfort in her chest. Chest computed tomography revealed a cavity, infiltration, and a nodule. A smear test for acid-fast bacilli was positive, a *Mycobacterium avium* complex transcription reverse-transcription concerted (TRC) test was negative, and an Mtb TRC test was withheld because the internal control was negative. After diluting the specimens, the internal control tested positive, and the sample tested negative. We diagnosed pulmonary *M. malmoense* infection based on a culture test. In conclusion, attention should be paid to the concentration of bacteria in Mtb TRC test samples, ensuring that the internal control provides expected results.

## Introduction

*Mycobacterium malmoense* is a member of the non-tuberculous mycobacteria (NTM). It is the second most common pathogen after *Mycobacterium avium complex* (MAC) in northern Europe, but is rare in Japan. It causes pulmonary infection and cervical lymphadenitis. The recommended combination of antibiotics and duration of treatment are not well established [1]. Here, we report a case of pulmonary infection caused by *M. malmoense*, which was difficult to distinguish from *Mycobacterium tuberculosis* (Mtb) infection, even using a transcription reverse-transcription concerted (TRC) test. In this case, the Mtb TRC test was withheld because the internal control was negative. We could rule out Mtb infection by diluting the specimens to account for possible internal control inhibition reactions due to high bacterial concentrations.

## Case presentation

**Figure 1:**
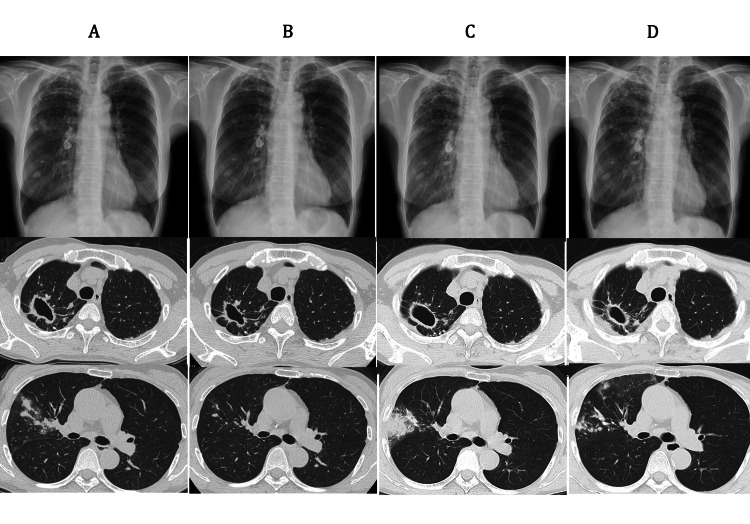
Chest roentgenogram and chest computed tomography (CT) (A) Disease onset: chest CT showed a cavity with a thickening of the wall in the right upper lobe, continuous infiltration from the right upper lobe to the middle lobe, and a nodule in the right lower lobe. (B) After the first treatment: a marked reduction in infiltration into the right lung was seen. The nodule in the right lower lobe remained unchanged. (C) At the time of recurrence: thickening of the cavity wall was seen, with increased infiltration into the right lung. (D) After 19 months of treatment: the infiltration had mostly resolved, but the shadows were more evident than after the first treatment. The nodule in the right lower lobe remained unchanged. This nodule was considered an old shadow.

A 64-year-old Japanese woman visited another hospital with complaints of bloody phlegm, a cough, and discomfort in her chest. A chest roentgenogram was ordered and results showed infiltration. The patient came to our hospital for further investigation. Chest computed tomography (CT) was done that revealed a cavity, infiltration, and a nodule in the right lung (Figure 1A).

**Figure 2:**
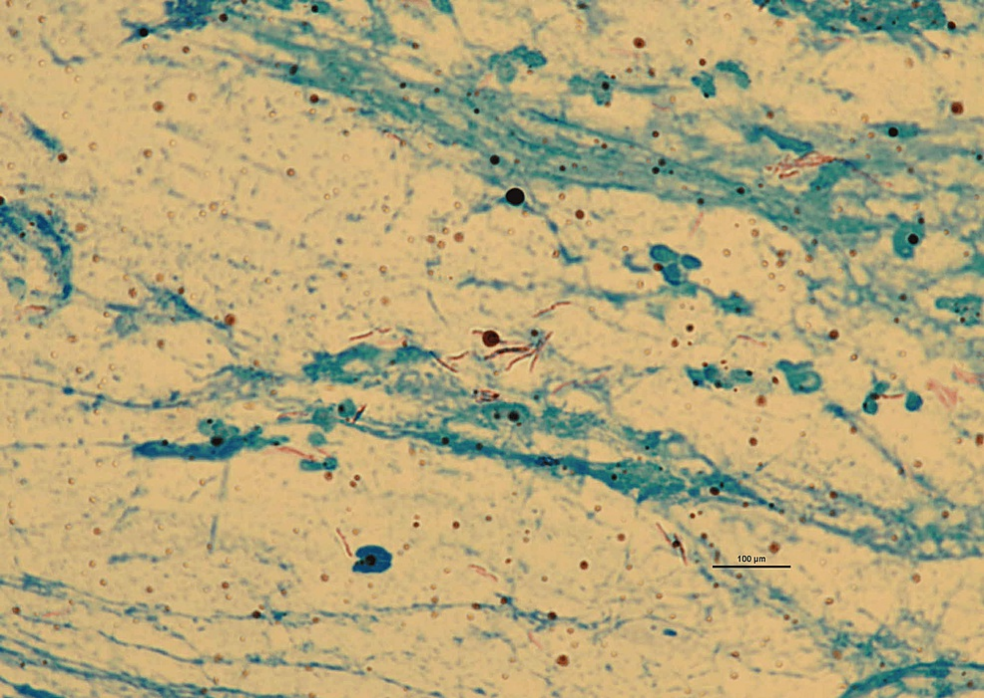
Acid-fast bacterium staining of the sputum in the gastric lavage (1000x)

The patient had a history of Mtb and hepatitis C, and had a 28 pack-year smoking history. Since Mtb infection occurred in childhood and at age 35, the details of her diagnosis and treatment were unknown. The patient worked at a gardening store and as a hospital cleaner. At presentation, her vital signs were as follows: body temperature, 36.3°C; percutaneous oxygen saturation, 98% on room air; and breath sound, no rales. The laboratory findings were as follows: C-reactive protein level, 0.01 mg/dL; white blood cell count, 6150/µL with 2.1% eosinophils; haemoglobin, 12.8 g/dL; and platelets, 23×10^3^/µL. Based on the clinical course, the imaging findings, and the clinical history, we suspected pulmonary tuberculosis and performed bacteriological examinations. The results showed a positive smear test for acid-fast bacilli (Figure 2).

The MAC TRC test (TRCReady®︎ MAC; Tosoh Corporation, Tokyo, Japan) was negative, and the Mtb TRC test (TRCReady®︎ MTB; Tosoh Corporation) was withheld because the internal controls were negative (Table 1).

**Table 1:** Results of bacteriological examinations

	Day 1 gastric lavage	Day 2 sputum	Day 3 sputum
Smear test for acid-fast bacilli	+	+	+
TRCReady®︎ MAC	˗	˗	N/A
TRCReady®︎ MTB (no dilution)	Withheld	Withheld	N/A
TRCReady®︎ MTB (no dilution, retesting)	Withheld	N/A	N/A
TRCReady®︎ MTB (diluted)	N/A	˗	˗
Taqman®︎ MTB	N/A	˗	N/A
Culture test	M. malmoense	M. malmoense	M. malmoense

Considering the possibility that an inhibitory reaction was occurring as a result of the high concentration of bacteria, we diluted samples and performed the TRCReady®︎ MTB test. The results were negative. To confirm this, we performed an outsourced Mtb polymerase chain reaction (PCR) test (Taqman®︎ MTB; Roche Diagnostics K.K., Tokyo), and the result was negative. A culture test identified *M. malmoense* through mass spectrometry.

The patient was diagnosed with pulmonary infection caused by *M. malmoense* and was treated with rifampicin (RFP), ethambutol (EB), and clarithromycin (CAM) according to the guidelines of the British Thoracic Society (BTS) [2]. Four months after the initiation of treatment, the eosinophil ratio had increased to 14.3%, and a skin rash with itching had appeared. The pulmonary symptoms and the CT findings were improved (Figure 1B). We discontinued treatment and the patient was placed on follow-up observation. Two and a half years later, a cough with sputum reoccurred, and CT findings worsened (Figure 1C). Culturing of the sputum led to the identification of *M. malmoense*. Treatment was resumed with RFP, EB, and CAM, and side effects such as itching and eosinophilia were managed using an anti-allergic agent. After eight months of treatment, results of a sputum smear test for acid-fast bacilli and a culture test came out negative. After 19 months of treatment, liver enzymes were found to be elevated and a liver examination was required. The clinical symptoms and CT findings were mostly improved (Figure 1D). Treatment was discontinued after 19 months, considering the possibility that the elevation of liver enzymes might be drug related.

## Discussion

Here, we have reported a case of pulmonary infection caused by *M. malmoense*. This case was difficult to distinguish from pulmonary Mtb, even using a TRC test. The clinical presentation of pulmonary *M. malmoense* infection involves a cough, dyspnea, fever, haemoptysis, and weight loss, and radiological features are the presence of a cavity and infiltration [1,3]. The main underlying diseases predisposing one to this infection are chronic obstructive pulmonary disease, bronchitis, emphysema, asthma, and old and healed Mtb infection [1,3].

To our knowledge, there are no previous reports on the difficulty in distinguishing NTM infection from Mtb infection by a TRC test in clinical practice. In our case study, the differential diagnoses were pulmonary tuberculosis and NTM infection because of the positive result of the smear test for acid-fast bacilli, clinical symptoms, imaging findings, and the history of pulmonary tuberculosis. It is important to correctly differentiate between Mtb and NTM to ensure appropriate treatment and, more broadly, public health management. In our case, the TRCReady®︎ MTB result was withheld because the internal control was negative, a phenomenon that has previously been reported [4]. In the report, when investigating the specificity of identification of the Mtb group by TRCReady® MTB, the internal control tested negative in 8 out of 147 NTM specimens including *M. malmoense*. Since the inhibition of the amplification process was considered a possibility, a diluted solution of *M. malmoense* was reassessed and a negative result for Mtb along with a positive result for the internal control was obtained. It was also mentioned in the report that the inhibition of amplification may occur in samples with concentrations of approximately 10^6^ CFU/mL or higher [4]. Since a strong positive in a smear test corresponds to 3.3×10^5^ CFU/mL or higher, caution should be taken when attempting to identify NTM from culture media [5].

Several limitations of this case study should be acknowledged. First, the patient was treated with a combination of antibiotics according to the BTS guidelines, but the recommended 12-month treatment period was not completed after testing negative for the bacterium, risking recurrence. Second, it is unknown when and how this patient became infected with *M. malmoense*. As far as we could find, there have been only three reports of pulmonary infections and one report of mediastinal lymphadenitis caused by *M. malmoense* in Japan since 2005 [6-9]. The prevalence of *M. malmoense* in Japan is unclear. To overcome these limitations, further studies are needed.

## Conclusions

We reported here a case of pulmonary infection caused by *M. malmoense*. In this case, it was difficult to distinguish it from pulmonary Mtb infection by a TRC test (TRCReady®︎ MTB). We ruled out Mtb infection by diluting the specimens to account for possible internal control inhibition reactions due to high bacterial concentrations.
